# Socioeconomic position over the life-course and subjective social status in relation to nutritional status and mental health among Guatemalan adults

**DOI:** 10.1016/j.ssmph.2021.100880

**Published:** 2021-07-21

**Authors:** Jithin Sam Varghese, Rachel Waford Hall, Ann M. DiGirolamo, Reynaldo Martorell, Manuel Ramirez-Zea, Aryeh D. Stein

**Affiliations:** a Nutrition and Health Sciences Program, Laney Graduate School, Emory University, Atlanta, GA, USA; b Hubert Department of Global Health, Emory University, Atlanta, GA, USA; c Georgia Health Policy Center, Georgia State University, Atlanta, GA, USA; d INCAP Research Center for the Prevention of Chronic Diseases (CIIPEC), Institute of Nutrition of Central America and Panama (INCAP), Guatemala City, Guatemala

**Keywords:** Perceived social status, Relative deprivation, Psychosocial framework, Subjective status, Psychological distress, Happiness, MacArthur ladder, BMI, Body mass index, FIML, Full Information Maximum Likelihood, INCAP, Institute of Nutrition of Central America and Panama, IQR, Interquartile Range, LMIC, Low- and middle-income country, MAR, Missing at Random, MI, Multiple imputation, SEP, socio-economic position, SRQ-20, World Health Organization Self-Reported Questionnaire-20, SSS, Subjective social status

## Abstract

**Objective:**

We study how life course objective socioeconomic position (SEP) predicts subjective social status (SSS) and the extent to which SSS mediates the association of objective SEP with nutritional status and mental health outcomes.

**Methods:**

We use data from participants of the INCAP Longitudinal Study 1969–2018 (n = 1258) from Guatemala. We use the MacArthur ladder for two measures of SSS - perceived community respect and perceived economic status. We estimate the association of SSS with health outcomes after adjusting for early life characteristics and life course objective SEP (wealth, schooling, employment) using linear regression. We use path analysis to study the extent of mediation by SSS on the health outcomes of body mass index (BMI; kg/m^2^), psychological distress (using the WHO Self-Reported Questionnaire; SRQ-20) and happiness, using the Subjective Happiness Scale (SHS).

**Results:**

Median participant rating was 5 [IQR: 3–8] for the perceived community respect and 3 [IQR: 1–5] for the perceived economic status, with no differences by sex. Objective SEP in early life and adulthood were predictive of both measures of SSS in middle adulthood as well as health outcomes (BMI, SRQ-20 and SHS). Perceived community respect (z-scores; 1 z = 3.1 units) was positively associated with happiness (0.13, 95 % CI: 0.07, 0.19). Perceived economic status (z-scores; 1 z = 2.3 units) was inversely associated with psychological distress (−0.28, 95 % CI: −0.47, −0.09). Neither measure of SSS was associated with BMI. Neither perceived community respect nor perceived economic status attenuated associations of objective SEP with health outcomes on inclusion as a mediator.

**Conclusions:**

Subjective social status was independently associated with happiness and psychological distress in middle adulthood after adjusting for objective SEP. Moreover, association of objective SEP with health was not mediated by SSS, suggesting potentially independent pathways.

## Highlights

• Subjective social status (SSS) is a self-appraisal of one's objective measures of socio-economic position (SEP; such as education and wealth) and social identity relative to their community.
• Perceived community respect and perceived economic status were positively associated with happiness and inversely associated with psychological distress respectively.
• Neither measure of SSS were associated with body mass index.
• The associations of objective SEP measures with health outcomes were not attenuated after adjusting for SSS, suggesting independent pathways.

## 1 Introduction

Objective measures of socioeconomic position (SEP), such as income, education, occupation and wealth, are important drivers of health outcomes (Glymour et al., 2014). Beyond their distal role as determinants of proximal behavioral and environmental risk factors, they are often considered as fundamental causes of health inequalities due to the resulting differential access to knowledge and resources (Link & Phelan, 1995). Individuals in low SEP are exposed to high levels of environmental and social stressors (Havranek et al., 2015). This increased exposure to stressors may affect health via psychological and biological responses which are greater in magnitude or duration (Cundiff et al., 2020). Subjective social status (SSS) is one's appraisal of objective SEP and their social identity relative to their community (Demakakos et al., 2018; Singh-Manoux et al., 2003, 2005a). SSS, as a result of social comparisons, reflects one's perception of their relative position. Relative deprivation (i.e. the negative subjective evaluation of one's socioeconomic position) over the life course and lower life satisfaction are associated with psycho-social stress, depressive thinking and physical health (Smith et al., 2012). SSS is hypothesized to mediate this association.

SSS was associated with both objective SEP measures and with health outcomes (Cardel et al., 2020; Cundiff et al., 2013; Habersaat et al., 2018; Hoebel & Lampert, 2020; Smith et al., 2019). However, associations of SSS with health is attenuated in most cases after adjusting for objective SEP measures such as household wealth (Demakakos et al., 2018; Ferreira et al., 2018; Hoebel & Lampert, 2020; Nobles et al., 2013; Prag, 2020; Scott et al., 2014; Shaked et al., 2016; Singh-Manoux et al., 2005b). SSS has been suggested previously as both a partial mediator of objective SEP and health as well as a distinct cause of health disparities based on observational studies (Brown-Iannuzzi et al., 2014; Hoebel & Lampert, 2020; Moreno-Maldonado et al., 2019). High SSS is associated with better self-reported health as well as biological and symptom-specific measures of health (Cundiff & Matthews, 2017; Matthews & Gallo, 2011; Zvolensky et al., 2015). However, current studies linking SSS and health outcomes suffer from a variety of epidemiological biases - uncontrolled confounding, measurement error (from common instruments) and unavailability of prospective life course data on SEP and health (Hoebel & Lampert, 2020; Li et al., 2018). Moreover, the transportability of results from one context to another is challenging, since reliability of the scale and consistency of the association vary by age, sex, race and region (Adler et al., 2008; Cundiff & Matthews, 2017; Demakakos et al., 2018; Giatti et al., 2012; Wolff et al., 2010a, 2010b). Given that SSS is easy to administer in population surveys, it is worthwhile to assess its usefulness as a predictor of different measures of health and well-being beyond standard objective SEP measures in low- and middle-income country (LMIC) settings.

Using data from a birth cohort (born during 1962–1977) from rural Guatemala, we first estimate associations of SSS based on economic standing and community respect with health outcomes measured in middle adulthood (from 2015 to 2018) after adjusting for objective SEP in early life and adulthood (Ramirez-Zea & Mazariegos, 2020; Stein et al., 2008). Second, we study how life course objective SEP predicts both SSS measures in middle adulthood. Third, we study the association of objective SEP over the life course with health outcomes in middle adulthood. Finally, we assess the extent to which the SSS measures mediates the associations of objective SEP over the life course with health in middle adulthood.

## 2 Methods

### 2.1 Study population

Data for this analysis come from the Institute of Nutrition of Central America and Panama (INCAP) cluster randomized trial in Guatemala. Four villages in the Department of El Progreso were selected, of which two were randomly assigned to receive Atole, a protein and moderate-energy drink and the other two to Fresco, a low-energy (no protein) drink. Both drinks had similar micronutrient content. Details of the supplementation and cohort characteristics have been described previously (Stein et al., 2008). The cohort consisted of 2392 children who were under 7 y of age at the launch of the intervention trial or were born during it. During the intervention trial period, children were followed up to age 7 years, death before the end of the intervention trial in 1977, or study end, whichever came first. All surviving cohort members were eligible for subsequent follow-up. The study has experienced considerable attrition, details of which have been reported previously, with 1386 (out of 2392) of the original cohort members residing in Guatemala successfully followed-up in middle adulthood (37–57 y) during the period from 2015 to 2018. The study villages have witnessed substantial changes from 1967 to 2018 in employment from agricultural (primarily subsistence farming) to non-agricultural wage labor and self-employment. Additionally, national efforts to improve schooling contributed to substantial improvements in literacy and schooling attainment (Melgar et al., 2020). These social and economic changes have led to rising standards of living over time (Varghese et al., 2021). The study population consists of cohort members who are residents of the original study villages and migrants to Guatemala city as well as other rural or urban areas.

### 2.2 Data collection and variable specification

#### 2.2.1 Objective socio-economic position

We created a harmonized wealth index using common assets (such as television, refrigerator and automobile) and housing characteristics (such as floor quality, water source and cooking medium) using Principal Components Analysis (PCA), the details of which were reported previously (Filmer & Pritchett, 2001; Varghese et al., 2021). The harmonized wealth index measures absolute gains in wealth over time using data collected in 1967, 1975, 1987, 2002, 2015–16, and 2017–18. The wealth index is computed as the weight computed for an asset (loadings) multiplied by the mean-standardized asset possession, which is then summed across all assets (Filmer & Pritchett, 2001). Attained schooling (in completed years), residence (rural or urban) and current employment status (formally employed vs informally employed/not seeking work/unemployed) of participants were collected in 2015–18.

#### 2.2.2 Subjective social status

The MacArthur ladder is a measure of subjective social status in which participants are asked to evaluate their social standing against a reference group (Singh-Manoux et al., 2003). Data on the MacArthur ladder for subjective social status were collected in 2017–18 (Singh-Manoux et al., 2003). Our analytic sample consists of those participants for whom the ladder measures were available from our original study population. Participants were shown a ladder which was to represent where people were positioned in their community. They were then asked to state their perceived position on the rungs of the ladder compared to others in their community where 1 was lowest and 10 was highest as per two questions:

a) *Economic status:* On a scale of 1–10, where 10 are the people who have more money and greater wealth and 1 are the people who have less money and less wealth, where would you place yourself?
b) *Community Respect:* On a scale of 1–10, where 10 are the people who have the most respectable position and the most respectable jobs in the community and 1 are the people with the least respectable or no work jobs. Where would you place yourself?

#### 2.2.3 Health outcomes in middle adulthood

Body mass index (BMI in kg/m^2^) was computed using height and weight as measured in 2015–16. General psychological distress, an indicator of mental health, was measured using the WHO Self-Reported Questionnaire-20 (SRQ-20), administered in 2017–18 (Beusenberg et al., 1994). The SRQ-20 is a screening tool consisting of 20 questions requiring dichotomous answers. For each participant, we summed the number of questions to which the participant responded in the affirmative (Range: 1 to 20). Global subjective happiness, an indicator of socio-emotional well-being, was measured using the Subjective Happiness Scale (SHS) administered in 2017–18 (Lyubomirsky & Lepper, 1999). The SHS consists of four items, rated on a Likert-type scale (1 – lowest, 5 – highest), on which participants are asked to rate themselves or compare themselves to others. We consider the average score for the four questions of SHS. Both SRQ-20 and SHS were administered in Spanish after translation. We did not conduct formal validation tests of the study instruments in our population, but both have been widely used and validated in other LMIC contexts (de QuadrosLde et al., 2015; Extremera and Fernández-Berrocal, 2014; Giang et al., 2006; Patel et al., 2008; van der Westhuizen et al., 2016). Although not formally validated in the Guatemalan context, these measures show expected associations based on literature. For example, the SRQ-20 and SHS are inversely correlated in our study (Spearman's rho: −0.23, p < 0.001). The original SHS scores items on 1–7 scale while the administered version scores items on 1–5 scale.

#### 2.2.4 Early life characteristics

Information on maternal characteristics (age in years, schooling), atole supplementation in village (yes or no), exposure to supplementation in first 1000 days (given its importance as a sensitive period), sex and birth year were collected at enrollment. We imputed maternal characteristics with the mean of birth village among those for whom data was not available.

### 2.3 Statistical analysis

Our analytic sample consists of 1258 out of 1386 participants (from an original cohort of 2392) who were residents of Guatemala followed up in the period 2015–18 and who reported subjective social status. A flowchart of the analytic sample is presented in Supplementary Fig. 1.

Among the analytic sample, we used the 2015–16 asset index for those who participated (n = 1036) in that wave and the 2017–18 asset index (n = 222) for the remaining participants as a measure of wealth in middle adulthood. All continuous variables (maternal age, maternal schooling, birth year, attained schooling, household asset index) were standardized to have a standard deviation of 1 unit. We do not apply any other transformations to our variables.

#### 2.3.1 Multivariable regression for association of subjective social status with health

We used multivariable linear regression to study the association of subjective social status with health independent of objective SEP. We suffixed models with C and E to distinguish between models fit using perceived community respect and perceived economic status respectively. We estimated the crude association of each subjective social status with health outcomes (Models 1C, 1E). Next, we used multiple imputation with chained equations (MICE; 10 datasets, 50 iterations) including outcome variables (BMI, psychological distress as SRQ-20, and happiness as SHS), early life characteristics, objective SEP, and subjective social status to impute missing values under a missing at random (MAR) assumption (Kontopantelis et al., 2017). We then sequentially adjusted for early life characteristics (Models 2C, 2E), followed by the adult SEP measures; attained schooling, employment, asset index) and rural vs. urban residence (Model 3C and Model 3E). We assessed for heterogeneity by sex of observed associations between SSS and our health outcomes using linear contrasts.

We assessed robustness to unmeasured confounding by estimating e-values (on the risk ratio scale) which indicates the minimum magnitude of association which an unmeasured confounder needs to have with both SSS and the outcome to explain away the observed association between SSS and the outcome (VanderWeele & Ding, 2017). E-values are risk ratio approximations estimated as exponentiations of standardized effect sizes multiplied by 0.91. The method assumes that the continuous variable has a logistic distribution (which is similar to a normal distribution) and does not require additional assumptions (Michael Borenstein et al., 2009).

We repeated the multivariable regression using inverse probability of censoring weights for being alive in 2017–18 and for reporting the subjective social status as a sensitivity analysis for selection bias. To test whether the association of SSS with each health outcome was modified separately by atole supplementation status, objective wealth or rural residence in adulthood, we examined the statistical significance of the interaction term.

#### 2.3.2 Path analysis

**Fig. 1:**
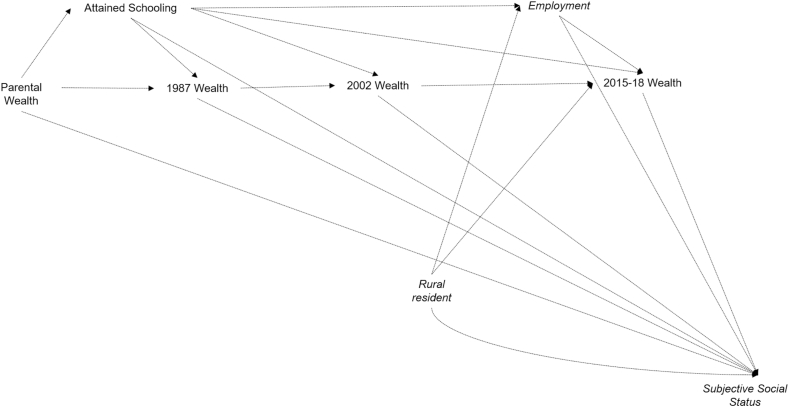
**Path diagram for association of objective socio-economic position and subjective social status** All associations were adjusted for early life characteristics (atole village, exposure in first 1000 days, maternal schooling, maternal age, sex, birth year, wealth in 1967–75). All objective socio-economic position (SEP) measures were adjusted for all previous objective SEP measures. Full path model is available in Supplementary Fig. 2. Subjective Social Status is Perceived Community Respect or Perceived Economic Status depending on the model.

We used path analysis to study how life course objective SEP predicts SSS as per Fig. 1 (full path model in Supplementary Fig. 2). We then fit the structural model to study the associations with health outcomes in middle adulthood. We fit the models on the analytic sample without mediation by SSS (Model *P1*), with community respect (Model *P2*) and with economic status (Model *P3*). We used Full Information Missing Likelihood (FIML) with robust standard errors under MAR assumption.

All analysis was carried out using R 3.5.1 using lavaan 0.6–6 (Rosseel, 2012) after adjusting for within-household clustering.

## 3 Results

Our analytic sample (n = 1258) was predominantly female (55 %), with a median age of 47 years and median attained schooling of 6 years (Table 1). Wealth, measured as harmonized household asset index (z-scores; mean ± standard deviation) increased over time from −3.6 ± 1.0 in 1967–75 to 1.9 ± 1.1 in 2015–18. Cohort members were employed both formally (48.8 %) and informally (41.4 %); most men were formally employed (64.0 %), while women were informally employed (46.6 %) or were not in the labor force (16.8 %). Median (25th and 75th percentiles) values for the MacArthur ladders for perceived community respect (Males: 6, IQR: 3–9; Females: 5, IQR: 2–8) and perceived economic status (Males: 3, IQR: 1–4; Females: 3, IQR: 1–5) were similar between men and women. Our analytic sample consisted mainly of non-indigenous populations currently living in rural Guatemala (73.1 %) but distribution of reporting SSS did not differ by sex or region of residence (Supplementary Fig. 3). Of the sample, 827 (65.7 %) lived in the original study villages and 204 (16.2 %) lived in Guatemala City. Additional descriptive statistics for pooled sample and stratified by men and women are presented in Table 1.

**Table 1:** Early life and life course socio-economic status characteristics of analytic sample (n = 1258).

	All (n = 1258)	Males (n = 561)	Females (n = 697)
Atole Village in 1969–77	52.5 %	51.9 %	53.1 %
Exposure in first 1000 days	40.8 %	42.1 %	39.7 %
Atole in first 1000 days	21.1 %	20.3 %	21.7 %
Maternal age	26 [21, 32]	26 [21, 32]	26 [21, 32]
	27.0 ± 7.1	27.1 ± 7.1	26.9 ± 7.1
Maternal schooling	1 [0, 2]	1 [0, 2]	1 [0, 2]
	1.3 ± 1.6	1.4 ± 1.6	1.3 ± 1.5
Birth Year	1970 [1967, 1974]	1970 [1967, 1974]	1970 [1967, 1974]
Attained schooling	6 [2, 6]	6 [3, 6]	4 [2, 6]
	5.0 ± 3.8	5.5 ± 3.8	4.5 ± 3.7
Household Asset Index			
1967–75	−3.6 ± 1.0	−3.6 ± 1.0	−3.6 ± 1.0
1987	−1.2 ± 1.5	−1.2 ± 1.4	−1.1 ± 1.5
2002	1.0 ± 1.1	1.0 ± 1.1	0.9 ± 1.1
2015–18	1.9 ± 1.1	1.9 ± 1.1	1.9 ± 1.1
Employment status			
Unemployed or not seeking work	9.8 %	1.1 %	16.8 %
Informal	41.4 %	34.9 %	46.6 %
Formal	48.8 %	64.0 %	36.5 %
Rural resident	73.1 %	74.5 %	71.9 %
Life Satisfaction	19 [17, 21]	20 [17, 21]	19 [17, 20]
Community Respect Ladder	5 [3, 8]	6 [3, 9]	5 [2, 8]
	5.7 ± 3.1	5.9 ± 3.0	5.5 ± 3.2
Economic Status Ladder	3 [1, 5]	3 [1, 4]	3 [1, 5]
	3.2 ± 2.3	3.0 ± 2.1	3.3 ± 2.5

Available sample is less than 1258 for: maternal age (n = 1249, Males: 555, Females: 694), maternal schooling (n = 1221, Males: 543, Females: 678), employment status (n = 1251, Males: 556, Females: 695), life satisfaction (n = 1244, Males: 552, Females: 692), wealth in 1987 (n = 853, Males: 419, Females: 434) and wealth in 2002 (n = 759, Males: 360, Females: 399). Summaries are in mean ± SD or median (25th-75th percentile) for continuous variables; percentage (%) for categorical variables.

Body mass index (BMI), 26.4 ± 4.1 kg/m^2^ among men and 29.2 ± 5.2 kg/m^2^ in women (Table 2). Median psychological distress (SRQ-20) for females (4, IQR: 2–7) was greater than for males (1, IQR: 0–3). Both males (4, IQR: 4–5) and females (4, IQR: 4–5) rated themselves high on happiness (Subjective Happiness Scale).

**Table 2:** Health outcomes of individuals from original study sample which were collected in 2015–16 and 2017-18.

	Pooled		Males		Females	
	n	Summary	n	Summary	n	Summary
Body mass index (kg/m^2^) in 2015–16	1017	28.1 ± 5.0	404	26.4 ± 4.1	613	29.2 ± 5.2
Psychological distress in 2017–18	1257	3 [1, 6]	561	1 [0, 3]	696	4 [2, 7]
		3.7 ± 3.8		2.3 ± 2.9		4.8 ± 4.1
Happiness in 2017–18	1244	4 [4, 5]	552	4 [4, 5]	692	4 [4, 5]
		4.1 ± 1.0		4.1 ± 0.9		4.0 ± 1.0

Psychological distress was measured using WHO SRQ-20; Happiness was measured using Subjective Happiness Scale. Mean ± SD or Median (25th-75th percentile) are reported for continuous variables; We excluded 6 pregnant women who had BMI measurements in the final analytic sample (Supplementary Fig. 1).

### 3.1 Association of subjective social status with health

Crude associations of perceived community respect suggested that higher perceived status was associated with lower psychological distress (−0.27, 95 % CI: −0.51, −0.03) and with higher happiness (0.16, 95 % CI: 0.10, 0.21). The association with happiness (0.13, 95 % CI: 0.07, 0.19) remained after adjusting for early life characteristics and objective SEP measures. Perceived economic status was associated positively with BMI (0.42, 95 % CI: 0.10, 0.75), was not associated with happiness (0.19, 95 % CI: −0.20, 0.58) and was associated inversely with psychological distress (−0.26, 95 % CI: −0.46, −0.06) before adjusting for other variables. After adjusting for early life characteristics and objective SEP measures, perceived economic status continued inversely associated with psychological distress (−0.28, 95 % CI: −0.47, −0.09). Results for crude and adjusted models are presented in Table 3.

**Table 3:** Association of subjective social status with health outcomes in middle adulthood (n = 1258).

	Perceived Community Respect			Perceived Economic Status		
	Model 1C	Model 2C	Model 3C	Model 1E	Model 2E	Model 3E
Body mass index (kg/m^2^) in 2015–16	0.04 (−0.32, 0.41)	0.09 (−0.25, 0.43)	0.05 (−0.28, 0.39)	0.41 (0.06, 0.76)	0.34 (−0.14, 0.81)	0.16 (−0.16, 0.48)
Psychological distress in 2017–18	−0.27 (−0.51, −0.03)	−0.13 (−0.36, 0.10)	−0.06 (−0.28, 0.17)	−0.26 (−0.46, −0.06)	−0.35 (−0.54, −0.16)	−0.28 (−0.47, −0.09)
Happiness in 2017–18	0.16 (0.10, 0.21)	0.15 (0.10, 0.21)	0.13 (0.07, 0.19)	0.19 (−0.20, 0.58)	0.13 (−0.05, 0.30)	0.09 (−1.70, 1.89)

Psychological distress was measured using WHO SRQ-20. Happiness was measured using Subjective Happiness Scale. Associations are displayed are multiple regression coefficients (95 % confidence interval; standard errors adjusted for multiple imputation); all continuous variables (maternal age, maternal schooling, birth year, attained schooling, household asset index) were standardized; BMI, SRQ-20 and Happiness were in original units of measurement. All models are suffixed with ‘C’ and ‘E’ for Perceived Community Respect and Perceived Economic Status respectively.

Model 1C, Model 1 E: Subjective social status.

Model 2C, Model 2 E: Adjusted for early life characteristics (atole village, exposure in first 1000 days, maternal schooling, maternal age, sex, birth year, wealth in 1967–75).

Model 3C, Model 3 E: Model 2 + attained schooling, wealth in 2015–18, employment (yes or no), rural residence.

Our bias analysis (e-value) for unmeasured confounding suggests that a hypothetical confounder with a risk ratio of 1.34 (lower confidence limit of 1.17) with perceived economic status and with psychological distress would be sufficient to explain the association of perceived economic status with psychological distress (Supplementary Fig. 4). Similarly, a hypothetical confounder with risk ratio of 1.51 (lower confidence limit of 1.34) with both perceived community respect and happiness could explain the observed association. The conversion of these risk ratios to comparable effect sizes (economic status with psychological distress: −0.31, community respect with SHS: 0.45) however indicate associations stronger than with wealth in middle adulthood (2015–18; from Model 3E – results not shown) for psychological distress (−0.23, 95 % CI: −0.45, −0.01) and happiness (0.17, 95 % CI: 0.11, 0.23).

Our results also indicated heterogeneity by sex for associations of perceived economic status with psychological distress. In women, 1 z-score increase in perceived economic status was associated with in −0.41 units (95 % CI: −0.66, −0.17) lower psychological distress among women but no change (0.01 units; 95 % CI: −0.28, 0.30) in psychological distress among men (Supplementary Table 1). We did not observe heterogeneity by sex for association of perceived community respect with any outcomes. We did not observe substantive heterogeneity by other variables, including exposure to the atole supplement, for association of perceived community respect or perceived economic status with health (Supplementary Table 2).

Results from the sensitivity analysis using inverse probability of censoring weights for being alive and reporting subjective social status were similar to unweighted results (Supplementary Table 3). Perceived community respect was associated with happiness (0.14, 95 % CI: 0.08, 0.19). However, perceived economic status was no longer associated with psychological distress (−0.19, 95 % CI: −1.32, 0.93).

### 3.2 Association of objective socio-economic position with subjective social status

Objective SEP measures in early life predicted later life objective SEP (Supplementary Table 4) after adjusting for early life characteristics. Objective SEP measures were also associated with BMI, psychological distress and happiness in middle adulthood after adjusting for early life characteristics and other SEP measures (*Model P1,* Table 4). Objective SEP measures over the life course were positively associated with both subjective social status measures though effect sizes were small (Model *P2* and *Model P3*; Table 4). For example, one z-score increase in attained schooling (0.12, 95 % CI: 0.05, 0.18), being a rural resident (0.12, 95 % CI: −0.02, 0.23), formal employment (0.18, 95 % CI: 0.06, 0.30) and one z-score increase wealth in 2015–18 (0.09, 95 % CI: 0.01, 0.16) were associated with an increase in community respect. One z-score increases in attained schooling (0.10, 95 % CI: 0.03, 0.16), wealth in 1987 (0.08, 95 % CI: 0.01, 0.15) and wealth in 2015–18 (0.16, 95 % CI: 0.09, 0.24) were associated with an increase in perceived economic status.

**Table 4:** Direct effects before and after accounting for mediation by subjective social status from path analysis (n = 1258).

	Independent variables							
	Wealth in 1969–77	Schooling	Wealth in 1987	Wealth in 2002	Rural resident	Employed	Wealth in 2015–18	SSS^a^
***Model P1***								
Body mass index (kg/m^2^) in 2015–16	−0.14 (−0.49, 0.22)	−0.17 (−0.57, 0.23)	0.03 (−0.42, 0.47)	−0.08 (−0.56, 0.39)	−1.05 (−1.76, −0.34)	−0.05 (−0.69, 0.59)	0.73 (0.38, 1.08)	
Psychological distress in 2017–18	−0.04 (−0.28, 0.20)	−0.54 (−0.80, −0.29)	0.40 (0.10, 0.70)	−0.31 (−0.64, 0.02)	−0.84 (−1.35, −0.33)	0.01 (−0.44, 0.47)	−0.17 (−0.42, 0.08)	
Happiness in 2017–18	−0.00 (−0.06, 0.05)	0.06 (0.01, 0.12)	−0.07 (−0.15, −0.00)	0.01 (−0.07, 0.09)	0.07 (−0.05, 0.19)	0.11 (−0.01, 0.22)	0.19 (0.12, 0.26)	
***Model P2***								
Perceived Community Respect (z-scores)	−0.05 (−0.12, 0.01)	0.12 (0.05, 0.18)	0.01 (−0.06, 0.08)	−0.05 (−0.14, 0.04)	0.12 (−0.02, 0.25)	0.18 (0.06, 0.30)	0.09 (0.01, 0.16)	
Body mass index (kg/m^2^) in 2015–16	−0.14 (−0.49, 0.22)	−0.17 (−0.56, 0.23)	0.03 (−0.42, 0.48)	−0.09 (−0.56, 0.39)	−1.05 (−1.76, −0.33)	−0.05 (−0.69, 0.59)	0.73 (0.38, 1.09)	−0.01 (−0.31, 0.28)
Psychological distress in 2017–18	−0.04 (−0.28, 0.19)	−0.54 (−0.79, −0.28)	0.40 (0.10, 0.70)	−0.31 (−0.64, 0.02)	−0.83 (−1.34, −0.32)	0.03 (−0.43, 0.48)	−0.17 (−0.42, 0.08)	−0.06 (−0.28, 0.17)
Happiness in 2017–18	0.00 (−0.06, 0.06)	0.05 (−0.01, 0.11)	−0.08 (−0.15, −0.00)	0.02 (−0.06, 0.10)	0.05 (−0.06, 0.17)	0.08 (−0.03, 0.20)	0.18 (0.11, 0.25)	0.13 (0.07, 0.18)
***Model P3***								
Perceived Economic Status (z-scores)	−0.01 (−0.07, 0.05)	0.10 (0.03, 0.16)	0.08 (0.01, 0.15)	0.02 (−0.06, 0.10)	−0.07 (−0.20, 0.06)	0.03 (−0.09, 0.14)	0.16 (0.09, 0.24)	
Body mass index (kg/m^2^) in 2015–16	−0.14 (−0.49, 0.21)	−0.19 (−0.58, 0.21)	0.03 (−0.42, 0.48)	−0.09 (−0.56, 0.39)	−1.04 (−1.75, −0.34)	−0.05 (−0.69, 0.59)	0.71 (0.36, 1.06)	0.12 (−0.19, 0.43)
Psychological distress in 2017–18	−0.04 (−0.28, 0.19)	−0.52 (−0.78, −0.27)	0.41 (0.11, 0.71)	−0.30 (−0.63, 0.02)	−0.86 (−1.37, −0.34)	0.02 (−0.44, 0.48)	−0.14 (−0.39, 0.11)	−0.21 (−0.40, −0.02)
Happiness in 2017–18	−0.00 (−0.06, 0.06)	0.06 (−0.00, 0.11)	−0.08 (−0.16, −0.01)	0.01 (−0.07, 0.09)	0.08 (−0.04, 0.19)	0.10 (−0.01, 0.22)	0.18 (0.10, 0.25)	0.09 (0.03, 0.14)

Associations are path analysis coefficients (95 % confidence intervals) as per path model (Model P1 to P3) in Fig. 1 adjusted for early life characteristics (atole village, exposure in first 1000 days, maternal schooling, maternal age, sex, birth year) using full information maximum likelihood (FIML) under missing at random. Wealth is measured as temporally harmonized wealth index measured during a survey year; all continuous variables (maternal age, maternal schooling, birth year, attained schooling, household asset index) were standardized.

a SSS – Subjective Social Status is Perceived Community Respect or Perceived Economic Status depending on the model. Psychological distress was measured using WHO SRQ-20; Happiness was measured using Subjective Happiness Scale. BMI, psychological distress and happiness were in original units of measurement.

### 3.3 Association of objective socio-economic position and subjective social status with health

Direct effects for objective SEP measures with the outcome were not attenuated after adjusting for either of the subjective social status measures. Results from the path analysis suggest neither perceived community respect (*Model P2*) nor perceived economic status (*Model P3)* were associated with BMI. One z-score increase in perceived community respect was associated with higher happiness (0.13, 95 % CI: 0.07, 0.18), consistent with results from linear regression (0.13, 95 % CI: 0.07, 0.19). Perceived community respect was not associated with psychological distress; however, perceived economic status was. One z-score increase in perceived economic status was associated with a decline in psychological distress (−0.21, 95 % CI: −0.40, −0.02) and an increase in happiness (0.09; 95 % CI: 0.03, 0.14).

## 4 Discussion

Our results show that objective SEP in early life and adulthood influence subjective social status in this cohort of rural-born non-indigenous adults in Guatemala. Attained schooling and cross-sectional wealth were associated with both perceived community respect and perceived economic status. In this community, well-respected include being teachers with permanent jobs, nurses, doctors, midwives, private sector jobs with a good income, and public sector jobs with a government entity. Results from our analysis suggest that subjective social status has small but independent associations with health outcomes (perceived economic status with psychological distress and perceived community respect with happiness) in middle adulthood. These associations remained after adjusting for early life characteristics and life course measures of objective SEP, consistent with results from studies in other settings (Adler et al., 2000; Prag, 2020). Direct effects of objective SEP measures with psychological distress (as measured by SRQ-20) and happiness (as measured by SHS) were not attenuated with the addition of SSS as a mediator. Gains in objective SEP alone, without improvement in SSS (or its mediating pathways), may be insufficient for better mental health.

Research from a Swedish cohort in middle-to late-adulthood near retirement suggests higher education and lower SSS were associated with depressive symptoms, results contrary to our findings for association of attained schooling with psychological distress (Nyberg et al., 2019). The association of household wealth with psychological distress is also consistent with results from rural Uganda and Myanmar which found depression was associated with lower SSS and lower self-rated economic status but not cross-sectional wealth (Sasaki et al., 2021; Smith et al., 2019). These results are consistent with the relative deprivation hypothesis which suggests social comparisons and personal experiences may influence health and well-being, in addition to objective circumstances (Wilkinson & Pickett, 2007). We are unable to preclude inferences of bidirectional associations such that those who suffer from depression may rate themselves lower in SSS (reverse causality) which may then lead to further depressive symptoms.

Consistent with other studies on the association of material circumstances and socio-emotional well-being, we found that cross-sectional wealth is associated with happiness (Kahneman & Deaton, 2010; Killingsworth, 2021). Subjective social status may therefore reflect additional dimensions of well-being as suggested by its independent association with happiness by incorporating personal experiences as well as the role of social networks.

BMI was not associated with SSS after adjusting for objective SEP, consistent with results from cross-sectional studies in Mexico and East Asia (Fernald, 2007; Tang et al., 2016). However, results from England and USA suggest that lower SSS is associated with higher odds of obesity (Tang et al., 2016).

Randomized studies suggest robustness of subjective social status to temporary mood and chronic negative affect (Kraus et al., 2013; Pieritz et al., 2016; Schubert et al., 2016). Stress-reactive rumination (tendency to ruminate) and depressive cognitions (cognitive content related to depressive thinking) are potential pathways linking SSS to depression (Euteneuer, 2014; Schubert et al., 2016). The observed associations may be a result of feedback loops of social causation and health selection involving SSS, objective SEP and health status (Hoebel & Lampert, 2020). Research in Indonesian adults suggests that health status (comprising of self-rated health and activities of daily living) predicted subjective social status after seven years (Nobles et al., 2013). Our observed associations may therefore be a result of reverse causality and/or residual confounding though several longitudinal studies demonstrate an association of low SSS with deterioration in health (Euteneuer, 2014; Hoebel & Lampert, 2020; Subramanyam et al., 2012).

Our sensitivity analysis suggests that an unmeasured/uncontrolled confounder, stronger than the association of wealth with health outcomes in middle adulthood, could move our observed association of SSS with SRQ-20 and SHS to contain the null (VanderWeele & Ding, 2017). We use the association of wealth with health as a benchmark given its usefulness as a robust measure of socio-economic position in LMIC settings. Personality traits such as self-esteem, psychosocial factors such as life satisfaction or history of mental health issues such as depression may be such potential confounders (Hoebel & Lampert, 2020).

### 4.1 Limitations

Our study has many strengths including prospectively collected life course SEP data for over 50 years, two different measures of subjective social status and widely used measures for health assessment in a well characterized population. However, our study has some limitations. Firstly, neither SRQ-20 nor SHS have been validated in the ladino population of Guatemala. However, our results show strong associations in the directions expected from the literature (e.g. mental distress negatively associated with happiness; higher distress associated with lower SEP; Table 4), suggesting that these measures are working as intended. Moreover, BMI is a non-specific indicator of physical health status. Future research could explore the association of SSS with biomarkers for cardiovascular disease (such as triglycerides, HDL or waist circumference). Second, though the community was defined based on residence for this question, further research is required to determine if the participants evaluate themselves against those who also migrated from the community (Corcoran et al., 2011). Moreover, perceived community respect was associated with employment status and perceived economic status was associated with wealth after adjusting for other measures of objective SEP which suggests that the scales were valid. Third, our study population is representative of original residents of rural ladino villages in Guatemala who remained within the country. As a result, our findings may not be generalizable to indigenous communities nor those who emigrated from Guatemala to other countries. Finally, we assumed missing at random (MAR) and used multiple imputation or full information maximum likelihood procedures for our analysis. Though the MAR assumption cannot be proved in practice and our cohort experienced non-monotone missingness, we believe there were no measured systematic predictors of missingness which we could additionally adjust for. Additional limitations include assumptions of linearity of associations (including with dichotomous dependent variables), and no unmeasured confounding of all path coefficients reported (VanderWeele, 2016).

## 5 Conclusion

Our results suggesting that objective SEP (especially attained schooling and employment) could improve SSS is important in the light of SARS-Cov 2. Guatemala, like other LMICs since the onset of the pandemic, has witnessed disparities in access to schooling, substantial loss of employment and rise in food insecurity (Action Against Hunger, 2020; Casasola, 2020; Lustig et al., 2020). Remedial actions for lost opportunity such as increased investments in schooling, technical training and food security initiatives therefore ought to be prioritized in order to ameliorate the lagged long-term physical and mental health consequences of the COVID-19 pandemic.

Our findings are consistent with the view the SSS displays modest associations with psychological distress and happiness beyond objective SEP (Prag, 2020). Potential pathways for exploration through which SSS may be associated with these outcomes include the role of peer and community social networks, autonomous nervous system and endocrine system as well as compensatory or adaptive behaviors (Cundiff et al., 2020; Euteneuer et al., 2012; Hoebel & Lampert, 2020). Further research on whether SSS could be improved via interventions strengthening social safety nets also ought to be explored.

## Financial disclosure

None.

## Ethics approval and consent to participate

We obtained ethical approval from the Institutional Review Board of Emory University (Protocol 95960). All participants gave written informed consent before participation.

## Funding

This work was supported by 10.13039/100000865Bill and Melinda Gates Foundation OPP1164115. The sponsor had no involvement in conduct of research and preparation of article.

## Availability of data and materials

The datasets generated and/or analysed during the current study are not publicly available. There are ethical or legal restrictions on sharing a de-identified data set. We cannot anonymize the data from this cohort as all individuals come from one of four previously named villages and hence are readily re-identifiable once their demographic characteristics are known. We will not post data to a public archive, but we will make a replication data set available to bona fide researchers who agree to sign an LDUA and are covered under an IRB. Please contact the Research Center for the Prevention of Chronic Diseases (CIIPEC) at the Institute of Nutrition of Central America and Panama for requests. The local data protection manager is Dina Roche (email: droche@incap.int; phone: +502 5499 7220). The code is available at https://github.com/jvargh7/incap-sss-path-analysis.

## Author contributions

JSV: Conceptualization, Methodology, Formal analysis, Writing – original draft; RWH: Writing – review & editing; AMD: Writing – review & editing; RM: Writing – review & editing; MRZ: Data curation, Writing – review & editing; ADS: Conceptualization, Methodology, Writing – review & editing, Funding acquisition.

## Declaration of competing interest

None.
