# Supplementary material for: Socioeconomic position over the life-course and subjective social status in relation to nutritional status and mental health among Guatemalan adults
Source: SSM Popul Health. 2021 Jul 21;15:100880. doi: 10.1016/j.ssmph.2021.100880 (PMC8327130; doi:10.1016/j.ssmph.2021.100880)
Supplement: Multimedia component 1 [file mmc1.docx]

**ONLINE-ONLY SUPPLEMENTARY MATERIAL**

**Socioeconomic position over the life-course and subjective social status in relation to nutritional status and mental health among Guatemalan adults**

Jithin Sam Varghese, Rachel Waford Hall, Ann M. DiGirolamo, Reynaldo Martorell, Manuel Ramirez-Zea, Aryeh D Stein

**Contents**

[Supplementary Table 1. Sex-stratified association of subjective social status with health outcomes in middle adulthood (n = 1258) 2](#_Toc74084021)

[Supplementary Table 2. Heterogeneity in association of subjective social status with health outcomes in middle adulthood by wealth, atole supplementation and rural residence (n = 1258) 3](#_Toc74084022)

[Supplementary Table 3. Association of subjective social status with health outcomes in middle adulthood after inverse probability weighting for participation in 2017-18 (n = 1258) 4](#_Toc74084023)

[Supplementary Table 4. Direct effect of early life variables on life course socio-economic status measures (n = 1258) 5](#_Toc74084024)

[Supplementary Fig 1. Flowchart for participation 6](#_Toc74084025)

[Supplementary Fig 2. Path diagram for studying the association of subjective social status with health 7](#_Toc74084026)

[Supplementary Fig 3. Distribution of subjective social status by sex and region of residence 8](#_Toc74084027)

[Supplementary Fig 4. Directed acylic graph example for bias analysis from unmeasured confounding 9](#_Toc74084028)

# Supplementary Table 1. Sex-stratified association of subjective social status with health outcomes in middle adulthood (n = 1258)

|  |  | | |
| --- | --- | --- | --- |
|  | **BMI** | **SRQ-20** | **SHS** |
| **Perceived Community Respect** |  |  |  |
| In Females | -0.07  (-0.51, 0.37) | -0.13  (-0.45, 0.20) | 0.15  (0.08, 0.23) |
| In Males | 0.23  (-0.18, 0.64) | 0.04  (-0.23, 0.31) | 0.09  (0.01, 0.17) |
| *Difference* | 0.30  (-0.23, 0.83) | 0.17  (-0.23, 0.57) | -0.06  (-0.17, 0.05) |
| **Perceived Economic Status** |  |  |  |
| In Females | 0.01  (-0.42, 0.43) | -0.41  (-0.66, -0.17) | 0.15  (-0.14, 0.45) |
| In Males | 0.47  (-0.00, 0.94) | 0.01  (-0.28, 0.30) | 0.04  (-0.07, 0.14) |
| *Difference* | 0.47  (-0.10, 1.03) | 0.42  (0.03, 0.80) | -0.12  (-0.38, 0.14) |

Associations are displayed are multiple regression coefficients (95% confidence interval; standard errors adjusted for multiple imputation); all continuous variables (maternal age, maternal schooling, birth year, attained schooling, household asset index) were standardized; BMI, WHO Self-Reported Questionnaire-20 (SRQ-20) and Subjective Happiness Scale (SHS) were in original units of measurement.

Adjusted for early life characteristics (atole village, exposure in first 1000 days, maternal schooling, maternal age, birth year, wealth in 1967-75), attained schooling, wealth in 2015-18, employment (yes or no), rural residence

# Supplementary Table 2. Heterogeneity in association of subjective social status with health outcomes in middle adulthood by wealth, atole supplementation and rural residence (n = 1258)

|  | **Perceived Community Respect** | | | **Perceived Economic Status** | | |
| --- | --- | --- | --- | --- | --- | --- |
|  | **BMI** | **SRQ-20** | **SHS** | **BMI** | **SRQ-20** | **SHS** |
| Wealth x SSS | 0.00  (-0.28, 0.29) | 0.14  (-0.32, 0.60) | -0.02  (-0.08, 0.03) | -0.03  (-0.34, 0.29) | -0.14  (-0.58, 0.31) | -0.00  (-0.39, 0.38) |
| Atole supplementation x SSS | -0.03  (-0.69, 0.64) | -0.02  (-0.46, 0.43) | -0.04  (-0.15, 0.08) | -0.26  (-0.94, 0.42) | -0.20  (-0.57, 0.17) | 0.05  (-0.14, 0.23) |
| Rural residence x SSS | -0.50  (-1.20, 0.20) | 0.14  (-0.32, 0.60) | -0.06  (-0.18, 0.06) | 0.19  (-0.46, 0.85) | -0.14  (-0.58, 0.31) | -0.06  (-0.25, 0.14) |

Associations are displayed are multiple regression coefficients (95% confidence interval; standard errors adjusted for multiple imputation); all continuous variables (maternal age, maternal schooling, birth year, attained schooling, household asset index) were standardized. Psychological distress was measured using WHO SRQ-20; Happiness was measured using Subjective Happiness Scale. BMI, WHO Self-Reported Questionnaire-20 (SRQ-20) and Subjective Happiness Scale (SHS) were in original units of measurement.

Adjusted for early life characteristics (atole village, exposure in first 1000 days, maternal schooling, maternal age, birth year, wealth in 1967-75), attained schooling, wealth in 2015-18, employment (yes or no), rural residence

# Supplementary Table 3. Association of subjective social status with health outcomes in middle adulthood after inverse probability weighting for participation in 2017-18 (n = 1258)

|  | **Perceived Community Respect** | | | **Perceived Economic Status** | | |
| --- | --- | --- | --- | --- | --- | --- |
|  | **Model 1C** | **Model 2C** | **Model 3C** | **Model 1E** | **Model 2E** | **Model 3E** |
| Body mass index (kg/m2) | 0.09  (-0.27, 0.46) | 0.13  (-0.21, 0.47) | 0.10  (-0.24, 0.45) | 0.38  (0.01, 0.75) | 0.30  (-0.04, 0.65) | 0.16  (-0.18, 0.50) |
| SRQ-20 | -0.28  (-0.52, -0.05) | -0.13  (-0.36, 0.10) | -0.05  (-0.54, 0.43) | -0.29  (-0.50, -0.09) | -0.32  (-3.31, 2.67) | -0.19  (-1.32, 0.93) |
| SHS | 0.16  (0.10, 0.22) | 0.15  (0.09, 0.22) | 0.14  (0.08, 0.19) | 0.14  (0.08, 0.21) | 0.67  (-3.06, 4.40) | 0.11  (-0.24, 0.47) |

Associations are displayed are multiple regression coefficients (95% confidence interval; standard errors adjusted for multiple imputation); all continuous variables (maternal age, maternal schooling, birth year, attained schooling, household asset index) were standardized; Psychological distress was measured using WHO SRQ-20; Happiness was measured using Subjective Happiness Scale. BMI, WHO Self-Reported Questionnaire-20 (SRQ-20) and Subjective Happiness Scale (SHS) were in original units of measurement. All models are suffixed with ‘C’ and ‘E’ for Perceived Community Respect and Perceived Economic Status respectively.

Inverse probability weights were derived using early life characteristics (atole village, exposure in first 1000 days, maternal schooling, maternal age, sex, birth year, wealth in 1967-75).

Model 1C, Model 1C: Subjective social status

Model 2E, Model 2E: Adjusted for early life characteristics (atole village, exposure in first 1000 days, maternal schooling, maternal age, sex, birth year, wealth in 1967-75)

Model 3C, Model 3E: Model 2 + attained schooling, wealth in 2015-18, employment (yes or no), rural residence

# Supplementary Table 4. Direct effect of early life variables on life course socio-economic status measures (n = 1258)

|  | **Wealth in 1969-77** | **Schooling** | **Wealth in 1987** | **Wealth in 2002** | **Rural**  **resident** | **Employed** | **Wealth in 2015-18** |
| --- | --- | --- | --- | --- | --- | --- | --- |
| Maternal age (y) | 0.23  (0.06, 0.40) | -0.40  (-0.55, -0.24) | 0.22  (0.02, 0.43) | 0.15  (-0.03, 0.33) | -0.09  (-0.17, -0.02) | -0.08  (-0.15, -0.02) | 0.06  (-0.07, 0.20) |
| Maternal schooling (y) | 0.26  (0.12, 0.41) | -0.04  (-0.18, 0.11) | -0.08  (-0.22, 0.07) | -0.02  (-0.22, 0.17) | -0.03  (-0.09, 0.04) | 0.00  (-0.08, 0.08) | -0.02  (-0.17, 0.14) |
| Birth Year^1^ (y) | -0.25  (-0.45, -0.05) | 0.05  (-0.16, 0.25) | 0.10  (-0.11, 0.32) | 0.06  (-0.19, 0.31) | 0.02  (-0.08, 0.11) | 0.04  (-0.07, 0.14) | -0.17  (-0.38, 0.04) |
| Sex = Male | 0.03  (-0.05, 0.10) | -0.04  (-0.11, 0.02) | 0.10  (0.03, 0.17) | -0.03  (-0.09, 0.03) | 0.02  (-0.00, 0.05) | -0.01  (-0.03, 0.02) | -0.03  (-0.08, 0.02) |
| Attained schooling (y) | 0.18  (0.09, 0.26) | 0.23  (0.17, 0.29) | 0.15  (0.07, 0.24) | 0.02  (-0.06, 0.10) | -0.00  (-0.03, 0.03) | 0.00  (-0.02, 0.03) | 0.04  (-0.01, 0.10) |
| Household Asset Index (z-scores) |  |  |  |  |  |  |  |
| 1967-75 |  | 0.13  (0.06, 0.20) | 0.27  (0.19, 0.35) | 0.11  (0.04, 0.18) | -0.02  (-0.05, 0.01) | 0.01  (-0.02, 0.03) | -0.01  (-0.07, 0.05) |
| 1987 |  |  |  | 0.20  (0.11, 0.29) | 0.04  (-0.00, 0.09) | -0.00  (-0.04, 0.04) | 0.04  (-0.03, 0.11) |
| 2002 |  |  |  |  | 0.05  (-0.02, 0.11) | 0.06  (0.03, 0.10) | 0.45  (0.38, 0.51) |
| Rural resident |  |  |  |  |  | -0.20  (-0.26, -0.14) | -0.08  (-0.24, 0.08) |
| Employed |  |  |  |  |  |  | 0.16  (0.04, 0.27) |

All associations adjusted for supplementation type (atole village) and exposure in first 1000 days from Model P1; Wealth is measured as temporally harmonized wealth index measured during a survey year. All continuous variables (maternal age, maternal schooling, birth year, attained schooling, household asset index) are standardized; ^1^ Birth year is centered at 1962 and standardized

# Supplementary Fig 1. Flowchart for participation

**
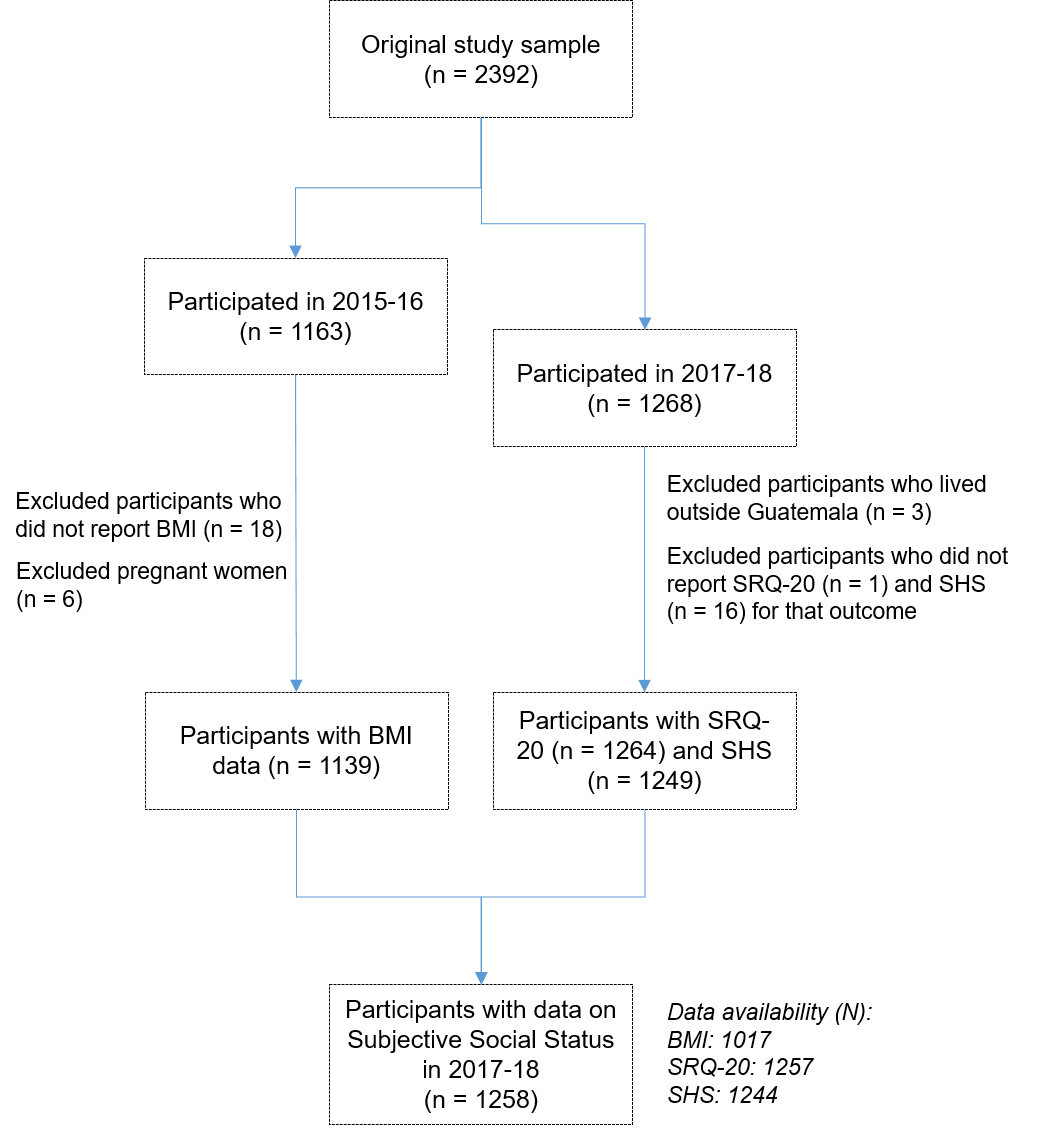
**

SRQ-20: WHO Self-Reported Questionnaire-20; SHS: Subjective Happiness Scale.

BMI was collected in 2015-16. SRQ-20 and SHS were collected in 2017-18.

# Supplementary Fig 2. Path diagram for studying the association of subjective social status with health


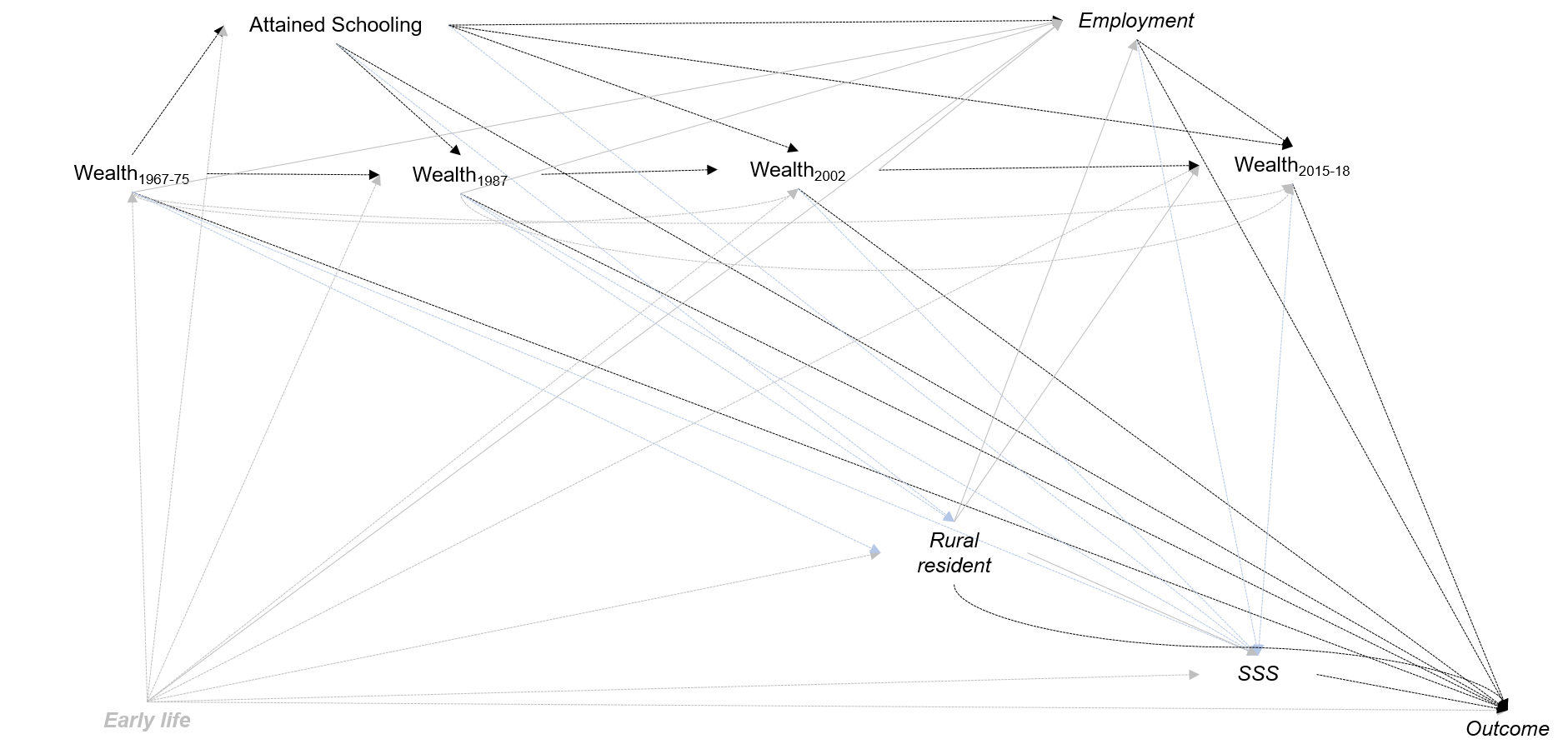


SSS: Subjective social status; Wealth as measured by temporally harmonized asset indices. Subjective Social Status is Perceived Community Respect or Perceived Economic Status depending on the model.

# Supplementary Fig 3. Distribution of subjective social status by sex and region of residence


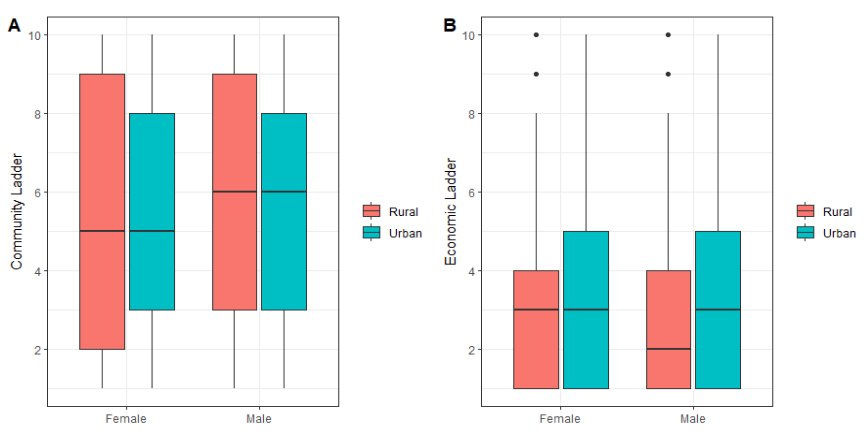


# Supplementary Fig 4. Directed acylic graph example for bias analysis from unmeasured confounding


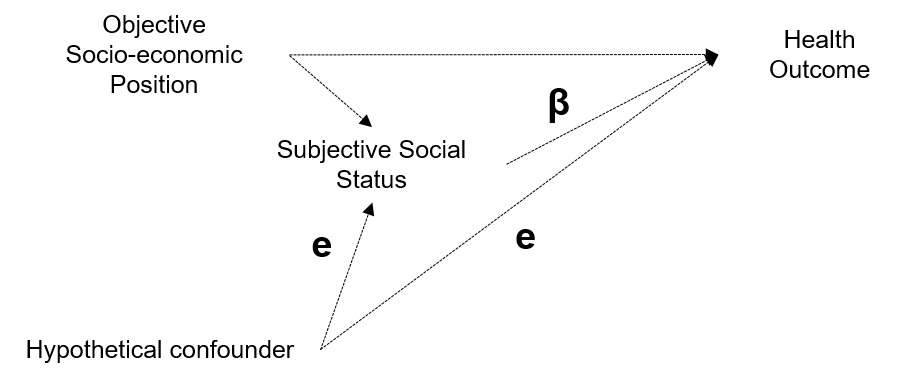


“e” is the e-value for unmeasured confounding or the minimum strength of association an unmeasured confounder should have with both the exposure (subjective social status) and outcome (such as BMI, psychological distress using WHO Self-Reported Questionnaire-20 and happiness using Subjective Happiness Scale) to nullify the observed association (β)
